# Standards Required for the Development of CDC Evidence-Based Guidelines

**DOI:** 10.15585/mmwr.su7101a1

**Published:** 2022-01-14

**Authors:** Vilma Carande-Kulis, Randy W. Elder, Dyann Matson- Koffman

**Affiliations:** 1 Office of Science Quality, Office of Science, CDC

## Abstract

CDC is the nation’s premier health promotion, prevention, and preparedness agency. As such, CDC is an important source of public health and clinical guidelines. If CDC guidelines are to be trusted by partners and the public, they must be clear, valid, and reliable. Methods and processes used in CDC guideline development should follow universally accepted standards. This report describes the standards required by CDC for the development of evidence-based guidelines. These standards cover topics such as guideline scoping, soliciting external input, summarizing evidence, and crafting recommendations. Following these standards can help minimize bias and enhance the quality and consistency of CDC guidelines.

## Introduction

In its role as the nation’s premier health promotion, prevention, and preparedness agency, CDC is an important source of public health and clinical guidelines. In the late 1950s, data from the first-ever disease surveillance program created by Alexander Langmuir were used to issue the first national guidelines for influenza vaccine (*1*). Since 1964, in partnership with the Advisory Committee on Immunization Practices (ACIP), CDC has issued recommendations on the use of vaccines for diseases in children, adolescents, and adults in the United States (*2*). CDC has published hundreds of evidence-based guidelines on a range of topics, from the prevention and control of infectious and noninfectious diseases and injuries to the promotion of environmental health and preparedness for natural and manmade disasters (*3*).

As a leader in domestic and global public health, CDC must produce guidelines that are clear, valid, and transparent. In 2011, CDC convened an internal work group of experts to establish standards and guidance for developing agency guidelines. Work group members included scientists, clinicians, and administrators throughout the agency. The work group adopted and adapted standards used by several guidelines authorities including the World Health Organization, the U.K. National Institute of Clinical Excellence, the U.S. Institute of Medicine, the U.S. Preventive Services Task Force (USPSTF), and the Community Preventive Services Task Force (CPSTF) (*4*–*8*). The work group created a “primer,” which describes the standard methods and processes that guideline developers are required to follow when developing CDC guidelines (*9*). This report summarizes the standards described in the primer and methodological developments since the primer was released.

## Definitions and Types of CDC Guidelines

A CDC guideline is any document issued under agency authority that contains recommendations for clinical practice or public health policy. Recommendations are statements that describe a specific prevention, treatment, or policy action. The scientific evidence underlying these statements is typically obtained through the systematic review of the literature and organized in evidence summaries. These evidence summaries present the causal associations that were critical to the decision-making process used to develop the recommendations (*10*). However, in certain cases, a large body of indirect evidence or factors, such as ethics, practical experience, feasibility or common sense, might strongly support a recommended action. These statements for recommended action have been referred to as good practice recommendations (*11*).

CDC guidelines fall under three general categories: interim, standard, and update. Interim guidelines are developed in response to emergencies, such as outbreaks and natural or manmade disasters. The term interim implies that CDC developed these guidelines using either expert opinion or indirect or emerging evidence, and the recommendations might change when more and better evidence becomes available. The *Guidance for Implementing COVID-19 Prevention Strategies in the Context of Varying Community Transmission Levels and Vaccination Coverage* is an example of an interim guideline (*12*). Standard guidelines consider the benefits and harms related to specific actions to address a disease, condition, or risk factor. In such guidelines, developers support evidence-based recommendations with systematic reviews of the literature. The 2016 *Guideline for Prescribing Opioids for Chronic Pain* is an example of a standard guideline (*13*). Updated guidelines replace or supplement previously published interim or standard guidelines, usually reporting on new evidence that results in one or more changes to the recommendations.

## Steps in Guideline Development

The following sections describe the standards for developing standard and updated CDC guidelines. Although these standards do not apply to interim guidelines, developers of such guidelines are encouraged to apply and adapt them to the extent feasible. Guideline standards are organized into six sequential steps: 1) assess the need for the proposed guideline; 2) determine guideline scope; 3) identify contributors, roles, and competing interests; 4) gather, summarize, and assess evidence; and 5) draft evidence-based recommendations. CDC’s Guideline Development and Reporting Checklist provides further details and examples of each standard (*14*).

### Assess the Need for the Proposed Guideline

Guideline development consumes considerable time and resources that should be allocated to producing original rather than duplicating existing credible guidelines. Duplication of guidelines, particularly if they contain dissimilar recommendations, will create confusion with the intended audience. Therefore, CDC requires guideline developers to use the CDC Guideline Development Decision Tool to determine the need for a proposed guideline and establish whether CDC is the most appropriate institution to lead such development (*15*). The tool uses the following questions to guide the user through the decision-making process:

- Will the guidelines address a current or potential public health burden or an emerging public health hazard?
- Is there a void in knowledge or practice that justifies the development of the guidelines?
- Do other guidelines on this topic exist?
- Does a literature base exist to support these guidelines?
- Have you determined from your intended audience or other stakeholders a need for new or updated guidelines on the topic?
- Does CDC have primary responsibility (or is CDC mandated by legislation, policy, or other directives) to lead development of these guidelines?
- If CDC is not responsible for leading the development effort, can a CDC partner develop the guidelines?
- Are adequate CDC resources available to develop the proposed guidelines?
- Is adequate time available to develop the guidelines?
- Is CDC able to publish, translate, distribute, and evaluate the guidelines?

### Determine Guideline Scope

**Affected Population:** Developers might categorize affected populations by age (e.g., newborn babies), behavior (e.g., men who have sex with men), geography (e.g., populations in states in the Pacific Northwest), occupation (e.g., health care workers) or other criteria.

**Intended Audience:** Public health guidelines often have diverse audiences (e.g., practitioners, policy makers, health care businesses, and government agencies); therefore, they will need to balance the information according to multiple needs.

**Guideline Setting:** Settings might include doctors or dental offices, hospitals, nursing homes, day care facilities, schools, colleges, workplaces, pharmacies, supermarkets, or the community at large. Settings might be narrowed by the populations they serve. For example, school-based guidelines might focus on schools in low-income areas, which could be determined by factors such as the proportion of children who are eligible for free school lunches.

### Identify Contributors, Roles, and Competing Interests

CDC guideline developers are encouraged to seek external input in the development of a guideline. The type and scope of a guideline affect the number of contributors and its complexity. Although interim guidelines might involve a relatively small group of internal agency experts, the development of standard guidelines might involve input from subject matter experts as well as individual and organizational stakeholders who might be affected by the implementation of the recommendations. Their inclusion in technical work groups and steering committees might help ensure the identification of potential issues that might affect guideline buy-in and adoption. Consultants and subject matter experts can contribute with input on selection of methods, interpretation of the evidence, and insight during the crafting of the recommendations. Other participants, such as official liaisons from stakeholder organizations, represent the views, concerns, and needs of their organizations and constituents.

The Federal Advisory Committee Act (FACA) provides the framework for consulting with experts outside the federal government to provide advice on appropriate recommendations (*16*). FACA delineates how federal advisory committees should be operated and managed. CDC’s webpage on management of federal advisory committees (*17*) describes how they legally operate and how members are nominated and selected. It also provides guidance on developing a charter and membership, conducting public meetings, issuing statements, using Federal Register announcements, keeping minutes, and documenting decisions. FACA also regulates the development of CDC guidelines that are not developed under the auspices of a federal advisory committee. In such cases, guideline developers are required to work with the CDC officials who manage federal advisory committee processes and procedures to make sure that external input is collected in compliance with FACA.

Users of CDC guidelines and recommendations need to know that financial, professional, or personal interests have not influenced the development of recommendations. A competing interest exists when professional judgment or actions concerning a primary interest, such as patients’ or the public’s welfare or the validity of research, might be improperly influenced by a secondary interest, such as financial gain, professional advancement, or personal relationships (*18*). Minimizing competing interests among contributors has been widely recognized as an important means to improve guideline scientific rigor, acceptability, and credibility (*19*,*20*). Competing financial interests might include research support, stock holdings, or employment at organizations affected by the guideline. Professional interests might include authorship of studies or provision of expert opinion publicly or in testimony related to the guideline topic. Personal and romantic relationships can interfere with subject matter experts’ judgment (*21*). CDC guideline developers must assess, disclose, and make every effort to either eliminate or manage interests that compete with the goals of producing unbiased, evidence-based recommendations (*22*). Ideally, group members involved in guideline development should have no employment or financial relation to a particular company that might unduly influence or give the appearance of influencing the guideline recommendations. Federal employees and special government employees (e.g., members of advisory committees) must abide by financial conflict-of-interest laws and regulations (*23*,*24*). Managing competing interests might require modifying roles of contributors. For example, someone with a direct financial interest in a recommendation might be required to abstain from becoming a member of the review team that conducts a systematic review or participating in the approval of the recommendation.

### Gather, Summarize, and Assess Evidence

Guideline developers establish trust with the reader by reporting on how the evidence was obtained, summarized, and evaluated in a thorough and transparent manner. Therefore, CDC developers of evidence-based guidelines must include sequential steps that 1) formulate review questions, 2) develop the search protocol, 3) select relevant literature, 4) abstract and summarize the evidence, and 5) assess evidence quality.

#### Formulate Review Questions

Guideline developers can use any appropriate framework for formulating the research questions for systematic reviews. The Population, Intervention, Comparison, Outcome (PICO) framework is one of the most frequently used (*25*) and is the basis for many variants that emphasize different aspects of the PICO elements (*26*–*31*). The Cochrane collaboration handbook provides useful guidance on how to develop research questions (*32*).

#### Develop the Inclusion Criteria and Search Protocol

Clear research questions make the development of the search protocol straightforward. Guideline developers are encouraged to work with a research librarian to develop a search protocol. They also are required to document the search protocol, preferably including specific terms used in each database searched. The Cochrane Collaboration handbook provides useful guidance on how to conduct a search for systematic reviews (*33*).

#### Select Relevant Literature

The way studies are selected might introduce substantial bias into the body of evidence. Selection of studies involves screening the search output according to the inclusion criteria. Typically, two reviewers independently screen titles and abstracts in a first stage, followed by independent screening of full papers that appear to meet the inclusion criteria. Any difference of opinion is resolved between the two reviewers or with the help of a third reviewer under the guidance of the technical lead. Reviewers might use flowcharts, most commonly the Preferred Reporting Items for Systematic Reviews and Meta-Analyses (PRISMA) flow diagram (*34*), to illustrate the process and software programs to manage the search output. CDC reviewers need to document the study selection process in detail.

#### Abstract and Summarize the Evidence

CDC reviewers should summarize findings from primary studies in a tabular format (*35*), which allows for the identification of each study’s major characteristics, study design, and selected outcomes. CDC guideline authors are required to provide the evidence that support the recommendations either by publishing it with the guideline, in a website, or on request.

#### Assess Evidence Quality

Evidence quality is an expression of the level of confidence that an effect estimate is accurate. Guideline developers are responsible for selecting the most appropriate method on the basis of what is feasible and useful for their needs. The most commonly used method to assess the quality of evidence mostly from clinical trials that use the Randomized Controlled Trial study design is Grading of Recommendations Assessment, Development and Evaluation (GRADE) (*36*). This approach determines evidence quality using several factors, including study design, limitation of the studies in execution and analyses, consistency of results across studies, applicability of the evidence to the populations and settings proposed for the intervention, and precision in the estimate of effect (*37*). The full body of evidence is given a quality rating of high, moderate, low, or very low depending on the degree of confidence that the true effect lies close to that of the estimate of effect, or alternatively the degree of confidence that the effect falls within a range that warrants the associated recommendation. In GRADE, evidence quality does not always equate with the strength of the recommendation. Although high-quality evidence usually leads to a strong recommendation, low-quality evidence can sometimes lead to strong recommendations depending on factors such as values, preferences, and costs (*38*).

Other guideline authorities, such as ACIP, USPSTF, and CPSTF, have adapted GRADE methods or developed alternative methods that are suitable for the type of evidence they deal with (*7*,*8*,*39*). Regardless of the method used to determine evidence quality, the guideline document must report in an explicit manner the quality of the evidence supporting the recommendations.

### Draft the Evidence-Based Recommendations

The development of evidence-based recommendations involves using a body of evidence as the scientific backbone behind a recommendation. It should also consider well-established clinical or public health principles and anticipate adverse outcomes that most likely would happen if the recommendation is implemented (*40*). In addition to being informed by the most appropriate and available evidence, recommendations should be clear, practical, set within a framework that acknowledges a range of social judgment, and reflect the views and experiences of both those being advised to act and the persons who might be affected by the action (*5*). When developing recommendations, CDC guideline developers should consider the benefits and harms reflected in the scientific evidence in the light of critical contextual factors such as feasibility, values, and preferences. The process will be influenced by value judgments, policy considerations, and assumptions about various factors.

## Conclusion

The standards described in this report are required for the development and reporting of CDC guidelines. Following these standards helps CDC guideline developers and their partners appropriately use evidence, minimize bias, and enhance quality and consistency. Observing these standards helps to make CDC guidelines worthy of the trust of the public and the health community. CDC standards and practices for developing guidelines will continue to evolve as advances in methods and technology offer the potential for more timely and useful guidelines. Machine learning has the potential to streamline time-consuming processes, such as searching for relevant evidence to inform guidelines (*41*). Living guidelines, which involve a continuous process of monitoring for new evidence that triggers updates when warranted, can help ensure that changing evidence or contexts are quickly reflected in updated guidelines (*42*). Computable guidelines, designed to be readily integrated into clinical decision support tools, can help ensure that clinical guidelines inform the decisions of health providers at the times they are needed (*43*). Such methods have already been incorporated in several specific CDC guidelines, such as those for opioid prescribing (*44*), and the lessons learned will serve as the basis for updates to guideline development standards.
